# Down-regulation of *acetolactate synthase* compromises *Ol-1*- mediated resistance to powdery mildew in tomato

**DOI:** 10.1186/1471-2229-14-32

**Published:** 2014-01-17

**Authors:** Dongli Gao, Robin P Huibers, Annelies EHM Loonen, Richard GF Visser, Anne-Marie A Wolters, Yuling Bai

**Affiliations:** 1 Wageningen UR Plant Breeding, P.O. Box 386, 6700 AJ Wageningen, The Netherlands; 2 Present address: Enza Zaden Beheer B.V., Haling 1E, 1602 DB Enkhuizen, The Netherlands

**Keywords:** Acetolactate synthase, *Oidium neolycopersici*, Resistance, *Solanum lycoperisum*, Amino acid homeostasis

## Abstract

**Background:**

In a cDNA-AFLP analysis comparing transcript levels between powdery mildew (*Oidium neolycopersici*)-susceptible tomato cultivar Moneymaker (MM) and near isogenic lines (NILs) carrying resistance gene *Ol-1* or *Ol-4*, a transcript-derived fragment (TDF) M11E69-195 was found to be present in NIL-*Ol-1* but absent in MM and NIL-*Ol-4*. This TDF shows homology to *acetolactate synthase* (*ALS*). ALS is a key enzyme in the biosynthesis of branched-chain amino acids valine, leucine and isoleucine, and it is also a target of commercial herbicides.

**Results:**

Three *ALS* homologs *ALS1*, *ALS2*, *ALS3* were identified in the tomato genome sequence. *ALS1* and *ALS2* show high similarity, whereas *ALS3* is more divergent. Transient silencing of both *ALS1* and *ALS2* in NIL-*Ol-1* by virus-induced gene silencing (VIGS) resulted in chlorotic leaf areas that showed increased susceptibility to *O. neolycopersici* (*On*). VIGS results were confirmed by stable transformation of NIL-*Ol-1* using an RNAi construct targeting both *ALS1* and *ALS2*. In contrast, silencing of the three *ALS* genes individually by RNAi constructs did not compromise the resistance of NIL-*Ol-1*. Application of the herbicide chlorsulfuron to NIL-*Ol-1* mimicked the VIGS phenotype and caused loss of its resistance to *On*. Susceptible MM and *On*-resistant line NIL-*Ol-4* carrying a nucleotide binding site and leucine rich repeat (NB-LRR) resistance gene were also treated with chlorsulfuron. Neither the susceptibility of MM nor the resistance of NIL-*Ol-4* was affected.

**Conclusions:**

ALS is neither involved in basal defense, nor in resistance conferred by NB-LRR type resistance genes. Instead, it is specifically involved in *Ol-1-*mediated resistance to tomato powdery mildew, suggesting that ALS-induced change in amino acid homeostasis is important for resistance conferred by *Ol-1*.

## Background

In their natural environment plants are constantly attacked by a variety of pathogens. Nevertheless, plants can detect and evade most infection attempts through constitutive and inducible immune responses. The inducible responses consist of two layers [1]. The first layer is triggered by multifarious pathogen-associated molecular patterns (PAMPs). The perception of PAMPs by plant pattern recognition receptors (PRRs) stimulates a number of cellular events, which include production of reactive oxygen species, activation of mitogen-activated kinases, enhanced expression of defense genes and production of antimicrobial compounds [2,3]. The second layer of inducible responses is activated by variable pathogen-specific effectors. Recognition of effectors by the plant is mostly mediated by a class of resistance proteins which contain nucleotide binding site and leucine rich repeat (NB-LRR) domains. The regulation and execution of both inducible responses involve hormone signalling pathways [4].

Emerging evidence illustrates that defense pathways are not only regulated by classical hormones, but also amino acid metabolic pathways constitute an important part of the plant immune system [5]. Besides the fact that some amino acids serve as precursors of antimicrobial compounds (e.g. glucosinolates) [6], amino acid homeostasis is pivotal for the outcome of plant-microbe interactions. A dominant nematode resistance gene in soybean encodes a serine hydroxymethyltransferase (SHMT), which plays a key role in one-carbon folate metabolism [7]. The SHMT allele in the resistant genotype encodes an isoform of the enzyme with altered kinetic properties compared with the isoform in susceptible genotypes. This altered SHMT enzyme is likely associated with perturbation of the folate pathway resulting in nutritional deficiency for nematodes. Overexpression of a pepper *asparagine synthetase* in Arabidopsis enhanced the resistance to bacterial and oomycete pathogens, which was correlated with increased asparagine levels [8]. Arabidopsis recessive downy mildew-resistant (*dmr1*) mutants defective in *homoserine kinase* were found to be resistant to the oomycete *Hyaloperonospora arabidopsidis* (*Hpa*). The resistance was homoserine-induced, and independent of known signalling pathways [9]. Suppression of the ortholog *SlDMR1* in tomato resulted in elevated resistance to powdery mildew *Oidium neolycopersici*[10]*.* Resistance to *Hpa* was also obtained in Arabidopsis *rar1-*suppressor (*rsp*) mutants, in which the level of threonine (Thr) was highly elevated [11]. The *rsp1* mutant carries a mutation in the *aspartate kinase2* gene [11], which catalyzes the first step in the aspartate-derived amino acid pathway. The *rsp2* mutant contains a loss-of-function allele of *dihydrodipicolinate synthase2*[11], which is the key enzyme in lysine biosynthesis. Disruption of an amino acid transporter *LHT1* confers a broad spectrum disease resistance in Arabidopsis plants, likely as a result of deficiency of glutamine [12].

*Oidium neolycopersici* (*On*) is an important biotrophic fungal disease for greenhouse crops. Unlike most powdery mildews that are host specific, *On* can infect a wide range of hosts, including species of the *Solanaceae* and *Cucurbitaceae* families [13]. A favourable strategy to control the disease consists of exploration of resistant alleles from wild species and introgression of these alleles into cultivated species to develop resistant cultivars. In tomato nine loci conferring resistance to *On* have been identified [14,15]. One of them - *Ol-1 -* originates from *Solanum habrochaites* G1.1560 [16], and confers incomplete resistance associated with slow hypersensitive response (HR) [17]. It is located on chromosome 6 [16,18] and has been fine-mapped to a region encompassing six predicted genes, based on the sequence of tomato cultivar Heinz 1706 [19] & unpublished results. None of the six genes encodes a protein with NB-LRR domains. Unravelling the identity of *Ol-1* has not been successful yet, because silencing of the predicted candidate genes individually did not attenuate the resistance level of the near-isogenic line carrying *Ol-1* (NIL-*Ol-1*) [unpublished results]. Another resistance gene - *Ol-4 –* which has been introgressed from *S. peruvianum* LA2172 confers complete resistance to *On* with fast HR [16]. It has been mapped to the *Mi-1* gene cluster on chromosome 6 [15]. Disease tests showed that NIL-*Ol-4* was resistant to root-knot nematodes, indicating the presence of a functional *Mi-1* homolog encoding a NB-LRR type protein. Furthermore, silencing of *Mi-1* homologs in NIL-*Ol-4* compromised the resistance to both *On* and root-knot nematodes, showing that *Ol-4* is a *Mi-1* homolog [20]*.*

In a previous study designed to elucidate the pathways of *On* resistance, a cDNA-AFLP approach was used to identify transcript-derived fragments (TDF) showing differential presence or intensity in resistant tomato NILs relative to susceptible Moneymaker (MM) after mock-inoculation or inoculation with powdery mildew *On*[17,21]. A BLAST analysis of the sequences of a number of differentially expressed TDFs was performed using the Sol Genomics Network (SGN) database to identify unigene sequences showing highest homology to each TDF. Subsequently, Tobacco Rattle Virus (TRV)-based Virus-Induced Gene Silencing (VIGS) constructs were generated targeting the unigenes. Then, VIGS was performed in the genotypes in which the TDF was detected to analyse whether silencing of targeted genes altered *On* resistance. In this way, it was shown that a putative *glutathione S-transferase* gene is required for *Ol-1*-mediated resistance against *On*[22].

In the present study we focused on another of these differentially expressed TDF (M11E69-195) and analysed its involvement in *On* resistance. M11E69-195 was specifically present in NIL-*Ol-1* but absent in MM and NIL-*Ol-4*[17,21]. The sequence of this TDF showed homology to *acetolactate synthase* (*ALS*). ALS (EC 2.2.1.6) is more frequently referred to as acetohydroxyacid synthase (AHAS) [23] in other studies. In this study, we describe it as ALS based on the annotation in the SGN database. ALS catalyzes the first step in the production of the branched-chain amino acids (BCAAs) valine, leucine and isoleucine [24]. It is extensively studied since it is a target of commercially successful herbicides. Different herbicide molecules can block substrate access to the active site of the ALS enzyme [25]. Here, we report the involvement of *ALS* in *Ol-1-*mediated resistance to powdery mildew in tomato.

## Results

### Down-regulation of two *ALS* genes simultaneously compromises *Ol-1*-mediated resistance to powdery mildew *On*

**Figure 1:**
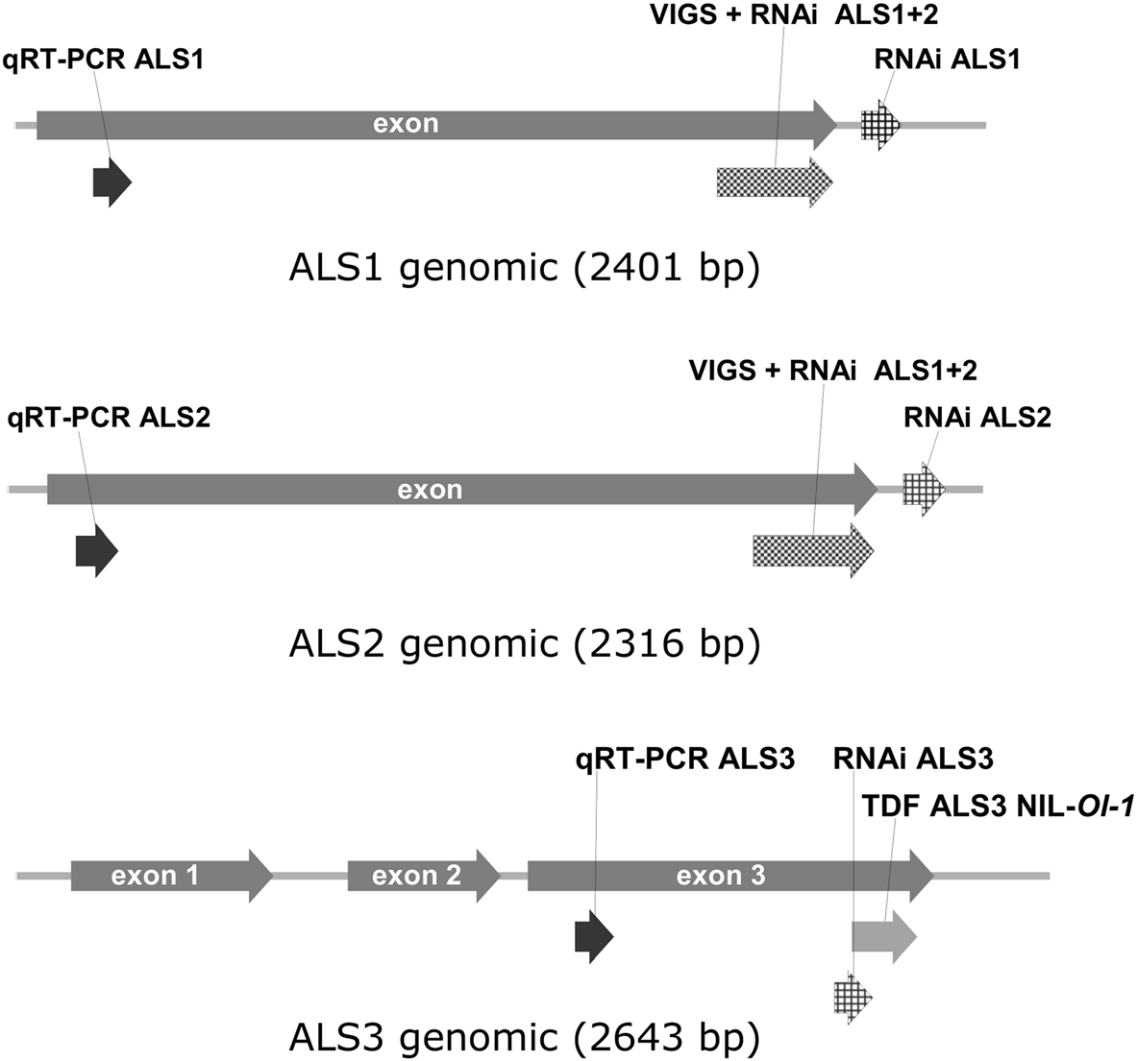
**Schematic representation of the genomic sequences of tomato *acetolactate synthase* genes *ALS1*, *ALS2 *and *ALS3*.** PCR fragments used in VIGS and RNAi constructs are indicated, as well as gene-specific fragments amplified in qRT-PCR analyses for quantification of gene expression. The fragment indicated as ‘VIGS + RNAi ALS1 + 2’ was present in TRV-U196237 and also used for stable transformation using an RNAi construct. TDF M11E69-195 from NIL-*Ol-1* showed the highest level of homology with exon 3 of *ALS3*.

**Figure 2:**
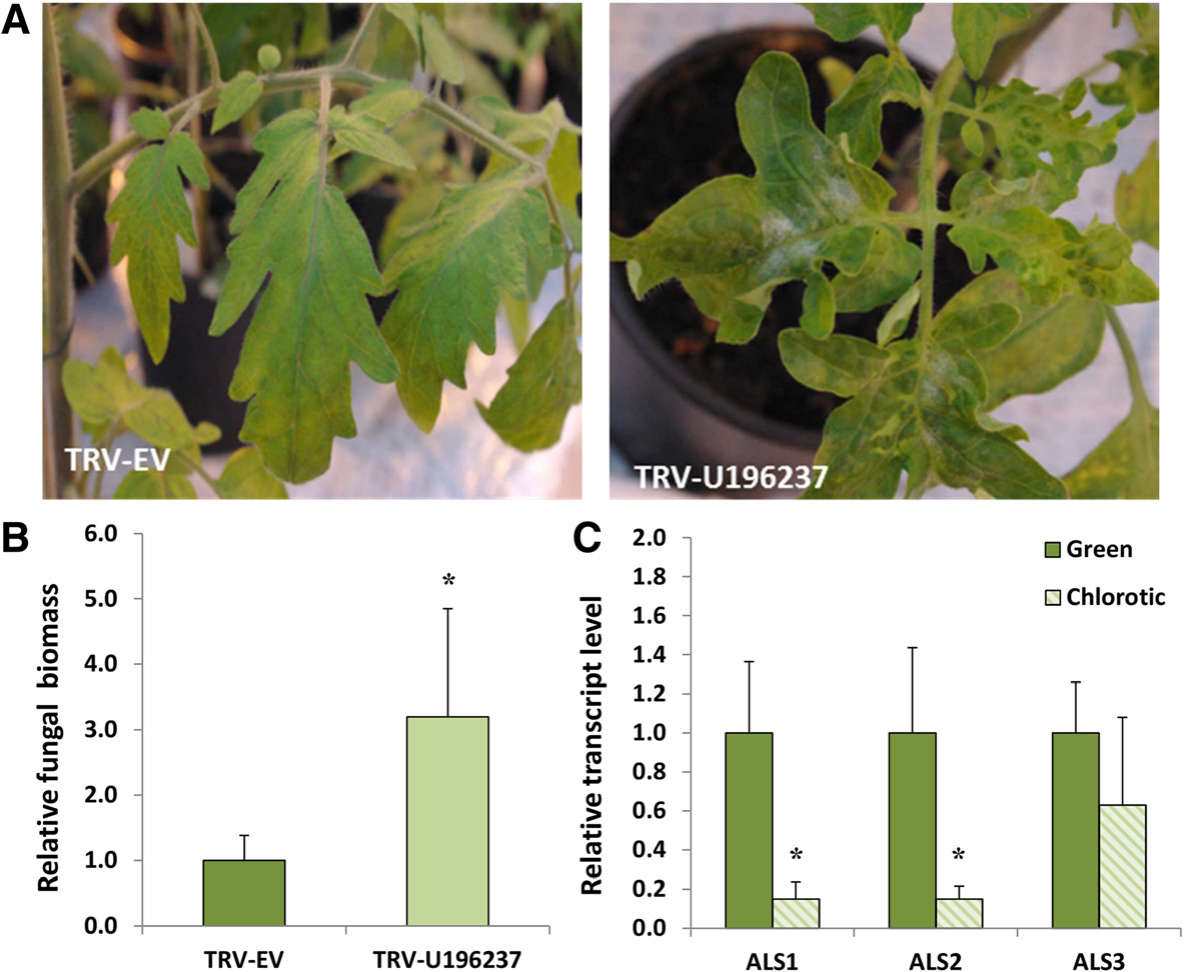
**Down-regulation of two *ALS *genes simultaneously via VIGS compromises *Ol-1*-mediated resistance. (A)**, Effects of VIGS on growth and *O. neolycopersici* infection of NIL-*Ol-1* plants infiltrated with empty vector as the control (TRV-EV) and TRV-U196237. **(B)**, Quantification of fungal biomass of TRV-EV plants and TRV-U196237 plants. Values were normalized relative to *elongation factor 1α* (*EF*), and calibrated to levels in TRV-EV plants. Error bars represent standard deviation of five TRV-EV and ten TRV-U196237 plants. For each plant DNA was extracted from pooled 3^rd^ and 4^th^ leaves. **(C)**, Transcript levels of *ALS1*, *ALS2*, and *ALS3* in green and chlorotic areas of TRV-U196237 leaves. Values were normalized relative to *EF*, and calibrated to levels in green area. Error bars represent standard deviation of five biological replicates. Asterisks indicate significant difference from the control according to independent-samples t-test (*P* <0.05).

In the cDNA-AFLP study by Li et al. [17,21] TDF fragment M11E69-195 (No. 24 in Appendix 1 in [17]; No. 71 in Appendix 1 in [21]) was observed to be present in *On*-resistant NIL-*Ol-1*, but absent in *On*-susceptible MM and *On*-resistant NIL-*Ol-4*. BLAST analysis of the sequence of this 195-bp TDF fragment was initially performed using the SGN database before the tomato genome sequence became publicly available. Highest homology was obtained for unigene SGN-U196237, a *Capsicum annuum ALS* gene (Additional file 1A). Primers were designed based on the U196237 sequence, and a 287-bp PCR product obtained using NIL-*Ol-1* cDNA as template was cloned into VIGS vector TRV2, resulting in vector TRV-U196237 (Figure 1). Infiltration of TRV-U196237 into NIL-*Ol-1* induced morphological changes, including short stature and curled leaves with chlorotic areas (Figure 2A). Subsequently, VIGS plants were inoculated with *On*. Quantification of fungal biomass showed that there was a significant (3-fold) increase on plants infiltrated with TRV-U196237 compared to plants infiltrated with the empty vector (TRV-EV) (Figure 2B).

After the tomato genome sequence became accessible a new BLAST analysis of the sequence present in the VIGS vector was performed. This resulted in the identification of three putative *ALS* genes in tomato named *ALS1* (*Solyc03g044330*), *ALS2* (*Solyc07g061940*) and *ALS3* (*Solyc06g059880*) (Additional file 1B). The latter one, although present on chromosome 6, does not reside in the *Ol-1* region [19]. ALS1 and ALS2 predicted proteins are 94% identical at the amino acid level, while ALS3 is quite different from ALS1 and ALS2 (75% and 78% identity with ALS1 and ALS2, respectively) (Additional file 1C). The *ALS1* and *ALS2* genes are predicted to contain one exon, whereas *ALS3* is predicted to contain three exons (Figure 1). Alignment of the TDF sequence (derived from the NIL-*Ol-1* line) with the three annotated *ALS* genes showed that the TDF was probably derived from the *ALS3* ortholog in *S. habrochaites (*Additional file 1A). However, alignment of the cloned fragment in the VIGS construct with the three annotated *ALS* genes resulted in highest homology to *ALS2*. This discrepancy can be explained, because for construction of the VIGS vector primers were designed based on the SGN unigene showing highest homology to the TDF, but no unigene based on EST sequences from *ALS3* was present in the SGN database. The alignment suggested that the VIGS vector targeted both *ALS1* and *ALS2*, but not *ALS3,* based on the assumption that an identical sequence of at least 21 nucleotides is necessary for efficient silencing. To validate the specificity of silencing, transcript levels of *ALS1*, *ALS2* and *ALS3* in NIL-*Ol-1* plants subjected to VIGS were measured by qRT-PCR using RNA isolated after pooling the third and fourth whole leaves of each plant. In this experimental set-up expression levels of the three *ALS* genes were not significantly reduced in TRV-U196237-infiltrated plants compared with TRV-EV-infiltrated plants (data not shown), although the alteration in leaf morphology indicated a VIGS effect. However, we noticed that fungal colony growth was stronger on the chlorotic areas of the leaves than on the green areas. Therefore, transcript levels of *ALS1*, *ALS2* and *ALS3* were compared between leaf samples collected from excised green and chlorotic areas of TRV-U196237-infiltrated plants. The expression levels of *ALS1* and *ALS2* were significantly lower in chlorotic areas in comparison with green areas, while expression of *ALS3* was also somewhat decreased in chlorotic areas, but not significantly (Figure 2C). This indicated that the fragment present in the TRV-U196237 VIGS construct specifically silenced *ALS1* and *ALS2*, but not *ALS3*.

**Figure 3:**
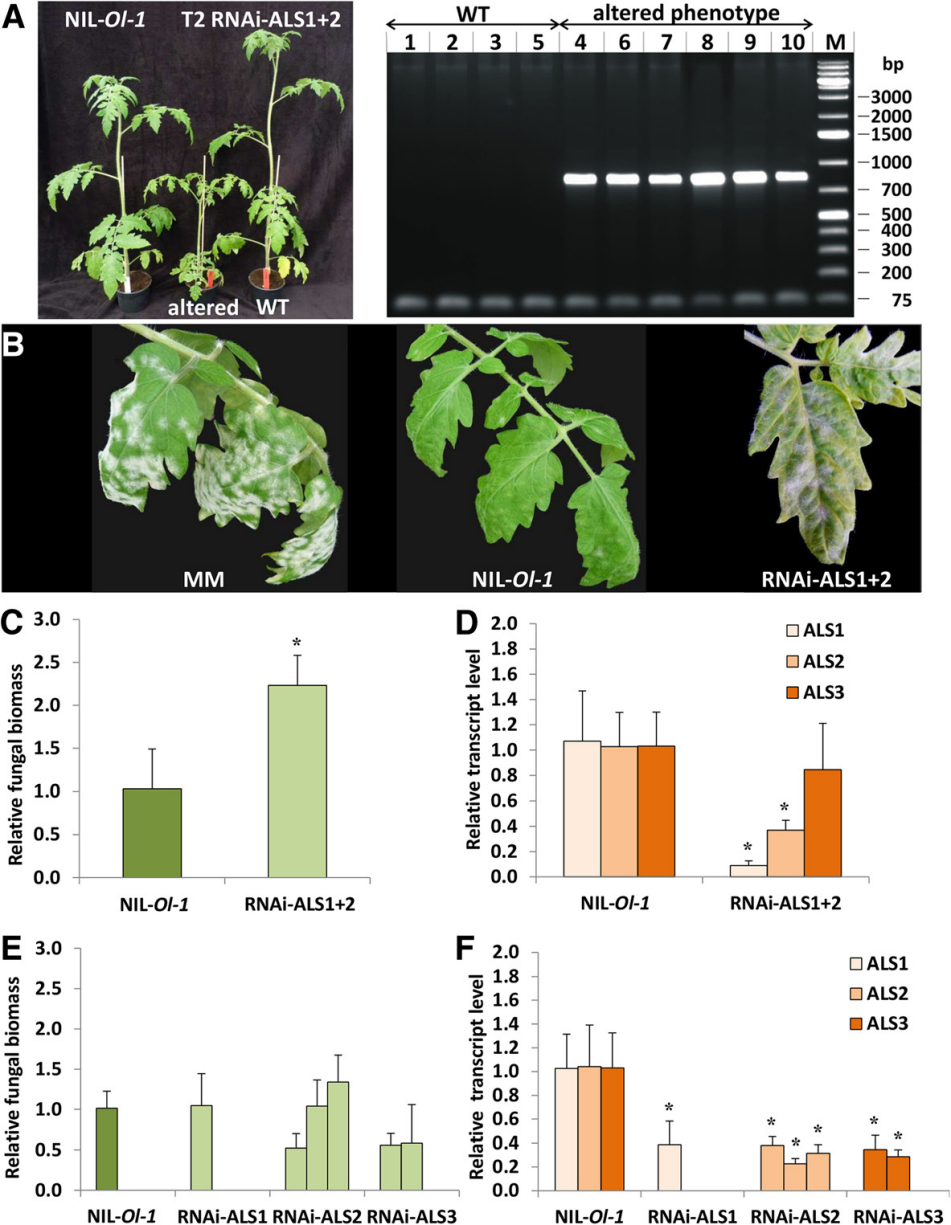
**Assessment of resistance to *Oidium neolycopersici *(*On*) in RNAi transformants of NIL**-***Ol-1*. (A)**, Segregation for phenotypic traits in T2 plants obtained after selfing RNAi-ALS1 + 2 transformant 216, compared with the NIL-*Ol-1* untransformed plant. PCR analysis of the 35S promoter of the silencing construct showed co-segregation of altered phenotype with presence of the expected PCR product. **(B)***On* symptoms on MM, NIL-*Ol-1* and NIL-*Ol-1* transformant obtained with the ALS1 + 2 RNAi construct (T2 plant). **(C)**, *On* fungal biomass and **(D)**, transcript levels of *ALS1*, *ALS2*, and *ALS3* in RNAi-ALS1 + 2 T2 plants of family 216 showing an altered phenotype, compared with that in NIL-*Ol-1* plants. **(E)**, *On* fungal biomass and **(F)**, transcript levels of *ALS1*, *ALS2*, and *ALS3* in NPTII-containing T2 plants in which three *ALS* genes were targeted individually by RNAi (RNAi-ALS1, RNAi-ALS2, and RNAi-ALS3). For each plant RNA was isolated from pooled 3^rd^ and 4^th^ leaves. Values were normalized relative to EF, and calibrated to the levels in untransformed NIL-*Ol-1* plants. Error bars represent standard deviation of three biological replicates. Asterisks indicate significant difference from the controls according to independent-samples t-test and one way analysis of variance (*P* <0.05).

To confirm the results obtained with VIGS, we generated stable transformants of NIL-*Ol-1* in which both *ALS1* and *ALS2* were silenced by an RNAi construct (RNAi-ALS1 + 2) containing an inverted repeat of the same sequence as present in the TRV-U196237 vector. We expected that when *ALS1* and *ALS2* were efficiently silenced by RNAi the transformants would show the same visible phenotype as in VIGS, *i.e.* smaller plants with chlorotic leaves. Nine primary transformants (T1) were selfed to produce T2 families. For each T2 family ten plants were phenotypically examined. One T2 family (216) showed clear segregation for the phenotypic traits mentioned above (reduced stature and chlorotic leaves) (Figure 3A). The altered phenotype co-segregated with the presence of the silencing construct, as indicated by amplification of the 35S promoter (Figure 3A). After inoculation with *On* the T2 plants with an altered phenotype showed increased sporulation when compared with the untransformed NIL-*Ol-1* plants (Figure 3B), although full susceptibility as in cultivar MM was not reached. Fungal growth and transcript levels of *ALS* genes were quantified in NIL-*Ol-1* plants and in T2 plants with altered phenotype. The results showed that the *On* fungal biomass in these T2 plants was significantly increased compared with NIL-*Ol-1* (Figure 3C). As expected, the gene expression levels of both *ALS1* and *ALS2* were significantly suppressed in the RNAi-ALS1 + 2 T2 plants, whereas *ALS3* expression was not significantly reduced (Figure 3D).

In addition to the production of stable transformants in which both *ALS1* and *ALS2* were silenced simultaneously, stable NIL-*Ol-1* transformants were produced in which the three *ALS* genes were silenced individually to evaluate their involvement in *Ol-1* resistance. No cross-silencing was observed (Additional file 2). Transformed T1 plants were selfed to obtain T2 families. One T2 family for *ALS1*, three for *ALS2*, and two for *ALS3* were obtained. The NPTII-containing, and thus transgenic, T2 plants were selected by PCR analysis. The transgenic T2 progeny showed a significant reduction of expression of the targeted *ALS* gene (Figure 3F). Silencing of the three *ALS* genes individually did not lead to morphological alteration, and fungal abundance was not enhanced compared to that in untransformed NIL-*Ol-1* plants (Figure 3E). The fact that suppression of individual *ALS* genes did not compromise *Ol-1*-mediated resistance, but suppression of at least two *ALS* genes compromised the resistance indicated that the function of *ALS* genes is likely overlapping.

We did not try to generate a construct targeting all three genes simultaneously, because no continuous stretch of at least 21 identical nucleotides is present when aligning the complete coding sequences of the three *ALS* genes (Additional file 1B).

### ALS is specifically involved in *Ol-1*-mediated resistance

**Figure 4:**
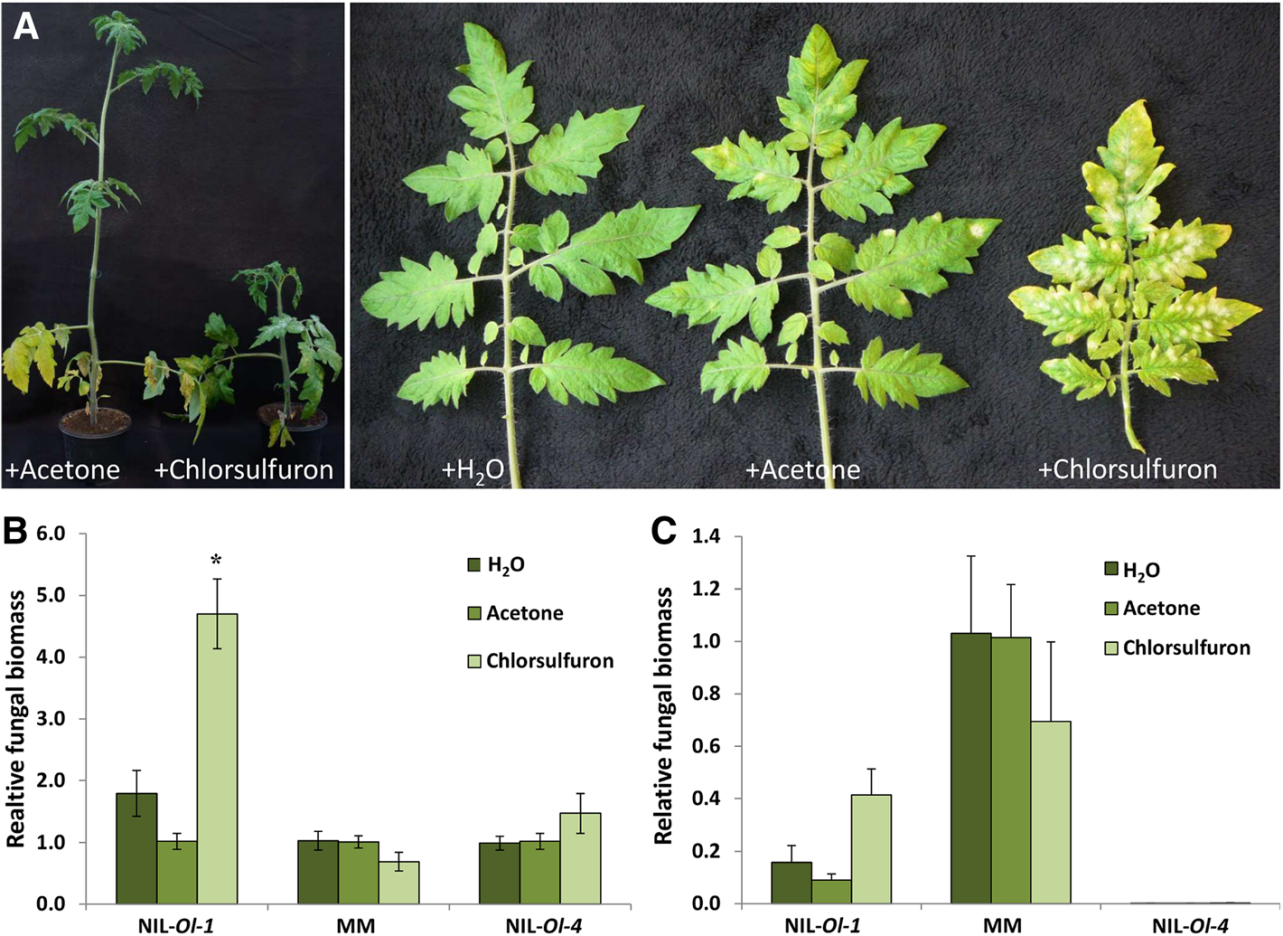
**ALS is specifically involved in *Ol-1*-mediated resistance against *Oidium neolycopersici *(*On*). (A)**, Phenotype of NIL-*Ol-1* plants in soil to which acetone (+ Acetone) or chlorsulfuron dissolved in acetone (+ Chlorsulfuron) has been added, and close-up of *On* development on the leaves. **(B)**, Relative *On* fungal biomass on NIL-*Ol-1*, Moneymaker (MM) and NIL-*Ol-4* plants grown in soil to which water (H_2_O), acetone or chlorsulfuron has been added. For each plant DNA was extracted from pooled 3^rd^ and 4^th^ leaves. Values are normalized relative to *EF*, and calibrated to level on plants grown in soil with acetone. Error bars represent three biological replicates for H_2_O and acetone treatments respectively, and 5 or more replicates for chlorsulfuron treatment. Two independent experiments were performed with similar results, and data from one experiment are presented. Asterisk indicates significant difference from the controls according to one way analysis of variance (*P* <0.05). **(C)**, Relative *On* fungal biomass on NIL-*Ol-1*, MM and NIL-*Ol-4* plants as in Panel B, but calibrated to the level on water-treated MM plants.

Acetolactate synthase is a well-known target for commercial herbicides, which block binding of substrates to the active site of the ALS enzyme [25]. We employed this system to determine whether ALS was generally involved in powdery mildew resistance or specifically involved in *Ol-1*-mediated resistance. The herbicide chlorsulfuron was used as the ALS inhibitor. First, we studied the effect of herbicide treatment in NIL-*Ol-1* plants. As chlorsulfuron was dissolved in acetone, we included plants to which only acetone was applied as well as plants to which water was applied as controls. The herbicide application caused inhibition of shoot growth and overall chlorosis of the plant (Figure 4A). Quantification of fungal DNA showed that a significant increase of fungal biomass was attributable to herbicide treatment, as compared with the acetone control (Figure 4A and B).

As powdery mildew fungi depend on living tissue for nutrient uptake, we wondered whether the probable perturbation of amino acid homeostasis due to silencing of *ALS* could be exploited by the pathogen, and in turn influence the basal defense. To address this question we treated susceptible tomato MM with chlorsulfuron. If ALS is important for basal defence against powdery mildew, one would anticipate an increase of sporulation. After herbicide treatment we observed morphological changes in MM plants, which were similar to those in NIL-*OL-1* plants. However, fungal biomass in chlorsulfuron-treated MM plants was similar to fungal biomass in water- and acetone-treated MM, suggesting that ALS was not involved in basal defence (Figure 4B). Chlorsulfuron was also applied to NIL-*Ol-4* plants to determine whether ALS is generally involved in powdery mildew resistance signalling pathways. Quantification of fungal biomass showed that herbicide-treated NIL-*Ol-4* plants retained a similar resistance level to powdery mildew as the control NIL-*Ol-4* plants, suggesting that ALS is dispensable for resistance conferred by *Ol-4*, encoding a NB-LRR type protein (Figure 4B).

Similar to the results obtained with the VIGS and RNAi plants, we observed that, although NIL-*Ol-1* plants in which ALS function was impaired showed increased susceptibility to *On*, full susceptibility as in cultivar MM was not reached. This is shown in Figure 4C, in which fungal biomass in *NIL-Ol-1* and *NIL-Ol-4* plants is calibrated to the level in water-treated MM.

### Expression of *ALS* genes upon powdery mildew attack in NIL-*Ol-1* and MM

**Figure 5:**
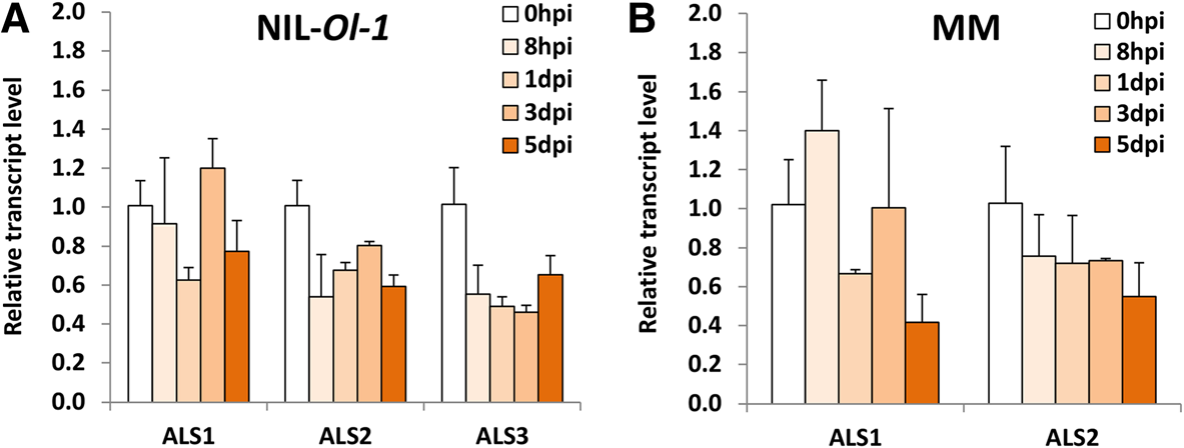
**Expression profiles of three *ALS *genes in tomatoes upon powdery mildew attack. (A)** NIL-Ol-1 and **(B)** Moneymaker (MM). Samples were harvested at 0 hpi (prior to inoculation), 8 hpi (hours post inoculation), 1 dpi (days post inoculation), 3 dpi and 5 dpi. For each biological replicate the 3rd and 4th leaves were pooled from three plants. Values are normalized relative to *EF*, and expression level at each time point after inoculation were calibrated to levels of counterpart plants without inoculation. Error bars represent standard deviation of three biological replicates. To test whether expressions of *ALS1* and *ALS2* were different between NL-*Ol-1* and MM, two-way between groups ANOVA was used.

To investigate the response of three *ALS* genes under powdery mildew attack, their transcript levels were measured in NIL-*Ol-1* and MM. Expression of *ALS1* and *ALS2* was detected in both genotypes, and they were not induced by powdery mildew inoculation in either genotype (Figure 5). ANOVA analysis indicated that there was no significant difference in expression levels of *ALS1* and *ALS2* between NIL-*Ol-1* and MM. *ALS3* expression was only detected in NIL-*Ol-1*, while in MM it may be either weakly expressed below detection level, or not expressed at all (Figure 5). We could exclude the possibility that primers for quantifying *ALS3* expression were not suitable for MM because a PCR product of the expected size was obtained using genomic DNA as template. Further, RNA-seq data of *ALS3* (*Solyc06g059880*) from tomato cultivar Heinz [26] confirm that *ALS3* is not expressed in tomato leaves (Additional file 3).

## Discussion

In a screen of differentially expressed transcripts showing a difference in presence or intensity when comparing powdery mildew-resistant NILs with susceptible MM, TDF M11E69-195 was identified [17,21], which shows homology to *acetolactate synthase*. This TDF was specifically present in NIL-*Ol-1*, but absent in both MM and NIL-*Ol-4*[17,21]. By targeting acetolactate synthases via VIGS, RNAi and herbicide application, we demonstrated that ALS activity was specifically important for *Ol-1-*based resistance (Figures 2B, 3C and 4B). ALS does not seem to be involved in basal defense as indicated by unchanged susceptibility after herbicide treatment of MM, nor required for resistance controlled by NB-LRR-type resistance genes as indicated by the results from herbicide treatment of NIL-*Ol-4* (Figure 4B).

### Possible involvement of *ALS3* in *Ol-1*-mediated resistance to *On*

The fact that TDF M11E69-195 was observed in NIL-*Ol-1*, but absent in both MM and NIL-*Ol-4* could be caused solely by the presence of nucleotide polymorphisms between MM and NIL-*Ol-1*. However, we have shown that the corresponding *ALS3* gene is truly differentially expressed, as *ALS3* transcripts were observed in leaves from NIL-*Ol-1* but not in MM leaves (Figure 5). The sequence of M11E69-195 from NIL-*Ol-1* showed higher similarity to *ALS3* than to *ALS1* and *ALS2* from *S. lycopersicum* (Additional file 1A). *ALS3* (*Solyc06g059880*) is located on the long arm of chromosome 6, but not in the *Ol-1* region. *ALS1* and *ALS2* are located on chromosomes 3 and 7, respectively. As NIL-*Ol-1* only contains (part of) chromosome 6 of *S. habrochaites* G1.1560 whereas all other chromosomes are from *S. lycopersicum* MM, we expected that the *ALS1* and *ALS2* genes from NIL-*Ol-1* were identical to those from MM. This was indeed observed after sequencing complete *ALS1* and *ALS2* cDNAs from NIL-*Ol-1* (data not shown). In contrast, sequencing of the complete *ALS3* cDNA from NIL-*Ol-1* revealed a number of SNPs and indels in NIL-*Ol-1* compared to the predicted sequence from tomato cultivar Heinz in the SGN database (Additional file 1B). NIL-*Ol-4*, containing an introgression of part of chromosome 6 from *S. peruvianum* accession LA2172 [19], was shown to contain *ALS1* and *ALS2* sequences identical to those from MM, whereas the *ALS3* sequence from NIL-*Ol-4* differed from both MM and NIL-*Ol-1*.

The VIGS and RNAi constructs targeted *ALS1* and *ALS2*, but not *ALS3* (Figures 2C and 3D). As *ALS1* and *ALS2* in all three genotypes are identical, but the effect of silencing is specific for NIL-*Ol-1,* we wonder whether *ALS3* plays a role in resistance to tomato powdery mildew conferred by *Ol-1*. Although *ALS3* is homologous to *acetolactate synthase* genes whose function has been proven, the exact function of the ALS3 protein is unknown. In plants ALS is a heteromultimer, consisting of catalytic and regulatory subunits [25,27,28]. All three tomato proteins ALS1, ALS2 and ALS3 are homologous to known catalytic subunits, such as the SuRA and SuRB proteins of *Nicotiana tabacum*[29]. In Solanaceous species from which genome sequences are available three *ALS* genes coding for catalytic subunits are present. In contrast, Arabidopsis only contains one *ALS* gene encoding the catalytic subunit, *i.e. At3g48560*.

In MM and NIL-*Ol-4* only *ALS1* and *ALS2* are expressed in leaves, while *ALS3* is not. Similarly, the orthologs of *ALS3* in *S. pimpinellifolium* and *S. tuberosum* are not expressed in leaves (Additional file 3). NIL-*Ol-1* is exceptional, as in this genotype *ALS3* is expressed in leaves, together with *ALS1* and *ALS2*, and therefore ALS3 may be incorporated in the ALS holoenzyme. Possibly, the presence of different catalytic subunits in the ALS holoenzyme confers different functionalities or substrate specificities. Although silencing of only *ALS3* in the NIL-*Ol-1* background did not result in increased susceptibility to *On* (Figure 3E), this does not exclude the possibility that *ALS3* is involved in resistance. The obtained transformants showed significant silencing of *ALS3* (Figure 3F), but no complete silencing comparable with a knock-out mutation was achieved. Additional experiments are needed to elucidate the function of *ALS3* in leaves of NIL-*Ol-1*, for example expression of the *S. habrochaites ALS3* gene in MM background.

### Involvement of amino acid homeostasis caused by altered ALS activity in *Ol-1-*mediated resistance

Although acetolactate synthase is a known target of several herbicides, it is unclear how herbicide-binding affects the amino acid metabolism in plants. Scheel and Casida [30] found that chlorsulfuron treatment of soybean suspension cultures caused a decrease of the valine and leucine contents, but had no effect on other amino acids. They showed that growth inhibition by chlorsulfuron was alleviated by supplying exogenous valine or leucine, or a combination of valine, leucine and isoleucine. Consistent with a reduction of BCAAs caused by an ALS-affecting herbicide, Ray [31] observed that addition of valine and isoleucine to excised pea root cultures reversed herbicide-induced growth inhibition. Growth retardation can also result from ALS feedback inhibition by individual end products. Chen et al. [28] showed that addition of valine or leucine to the growth medium inhibited root growth of Arabidopsis seedlings, whereas addition of isoleucine had no effect. When a combination of valine + isoleucine, or leucine + isoleucine was added to the medium root growth inhibition was less pronounced, suggesting isoleucine counteracted the inhibitory effect of valine and leucine [27]. Royuela et al. [32] detected an increase in the relative proportion of some amino acids other than BCAAs in chlorsulfuron-treated wheat and maize. Höfgen et al. [33] silenced *ALS* in potato by antisense inhibition, resulting in a decrease of ALS activity of up to 85%. Strong silencing of *ALS* resulted in severe growth retardation and stunting, and leaf chlorosis. Similar phenotypic alterations were obtained by treatment with an imidazolinone herbicide. Measurement of amino acids showed an accumulation of total free amino acids as well as perturbed composition in antisense and herbicide-treated plants. Unexpectedly, instead of decreased levels, elevated amounts of amino acids including valine, leucine and isoleucine were observed, especially in older sink leaves.

Recently, another example of a link between herbicide resistance, increased amino acid levels, and resistance to fungal species was reported. Patent US8383887 [34] discloses that corn plants expressing the bacterial *gdhA* gene (NADPH-dependent glutamate dehydrogenase) are resistant to aflatoxin accumulation following *Aspergillus* infection. Furthermore, corn and tobacco plants transformed with the *gdhA* gene are resistant to root rot following *Fusarium virguliforme* infection. Previously, it has been shown that tobacco plants transformed with the *gdhA* gene show an increased level of resistance to the herbicide glufosinate [35] and that total free amino acids were increased in these plants [36,37].

Taken together, the effect of ALS inhibition on levels of individual amino acids is difficult to predict, as it seems to depend on the level of residual ALS activity in different tissues, and the feedback-inhibition effect of (combinations) of individual amino acids. Despite this, we investigated whether BCAAs contents influence powdery mildew susceptibility or resistance in tomato cultivar MM and NIL-*Ol-1* by exogenously applying leucine, isoleucine and valine [10] & Additional file 4. Homoserine and threonine were also included in the experiment, because they were found to affect plant immunity, and threonine is the precursor of isoleucine. If a higher level of BCAAs contributes to *Ol-1*-mediated resistance, we expected to gain powdery mildew resistance to some degree in MM with elevated BCAAs levels. Quantification of fungal DNA showed that only exogenous application of homoserine significantly reduced the susceptibility of MM and increased resistance of NIL-*Ol-1* to *On*, whereas application of the other amino acids did not alter the responses of MM and NIL-*Ol-1* to *On*[10] & Additional file 4. We also did not observe the growth retardation which can be caused by individual end products, possibly because the concentration was not sufficiently high to cause this. The results suggested that instead of an elevated level, a reduced level of BCAAs or changed compositions of individual amino acids may play a role in *Ol-1*-mediated powdery mildew resistance. Amino acid deprivation is known to activate defences in Arabidopsis. For instance, the accumulation of camalexin, a pathogen-inducible antimicrobial phytoalexin was induced by BCAAs starvation [38]. An alternative hypothesis is the involvement of an amino acid-derived signal(s) in defense signalling pathways, as suggested for Arabidopsis genes *ALD1* and *AGD2* encoding aminotransferases [39]. Furthermore, studies on plant hormone conjugates showed that jasmonate (JA) can conjugate BCAAs [40] and, in particular, JA-isoleucine is the main bioactive form of the hormone [41]. In addition, altered expression of an enzyme involved in conjugation affects salicylic acid-mediated disease resistance [42].

In the case of *Ol-1*, perturbation of amino acid balance by silencing of *ALS* or herbicide treatment may impair the integrity of the signalling network, leading to the loss of resistance conferred by *Ol-1*. The unknown identity of *Ol-1* makes it harder to understand the link between *Ol-1-*mediated resistance and amino acid homeostasis. Cloning of *Ol-1,* determination of amino acid homeostasis, and dissection of changes in hormone signalling pathways will aid in understanding the requirement of ALS activity for *Ol-1*-based resistance and shed light on the interaction of amino acid metabolism and plant immunity.

## Conclusion

Tomato genome encodes three *ALS* genes. Silencing of each of them did not attenuate *Ol-1*-mediated resistance to tomato powdery mildew, while down-regulation of both *ALS1* and *ALS2* simultaneously or inhibition of the ALS activity resulted in the loss of *Ol-1*-mediated resistance to tomato powdery mildew. Further research on cloning of *Ol-1* and association of amino-acid homeostasis with ALS activity may provide insight into the role of amino acid metabolism in tomato resistance to powdery mildew.

## Methods

### Plant materials, fungal isolate and inoculation

All the near isogenic lines (NIL) have been described previously [15]. They were obtained by crossing wild tomato species containing the resistance gene(s) with *S. lycopersicum* cultivar Moneymaker (MM), three backcrosses with MM, followed by two selfings (BC3S2 plants). *Ol-1* was introgressed from *S. habrochaites* G1.1560, while *Ol-4* was introgressed from *S. peruvianum* (or *S. arcanum*) LA2172. *Oidium neolycopersici* (*On*) isolate Netherlands was maintained on susceptible MM plants in a growth chamber at 21/19°C (day/night). Fungal spores were washed off from heavily infected tomato leaves with tap water and diluted to a concentration of 2.5 × 10^4^ spores per mL. The inoculum was evenly sprayed on 3 to 4 weeks-old plants.

### Virus-induced gene silencing (VIGS)

VIGS was performed using the TRV-based vector system [43]. Primers for the TRV2 construct targeting SGN-U196237 were Fw-U196237-caccCAATGGGAGGATCGGTTCTA and Rv-U196237-ATCTCCCATCACCCTCTGT. A 290–bp fragment was amplified from cDNA of NIL-*Ol-1* plants, and subsequently cloned into pENTR/D-TOPO vector (Invitrogen). After verification of the sequence the fragment was introduced into the pTRV2-attR1-attR2 vector [43] via LR recombination. The resulting TRV-U196237 vector was transformed into *Agrobacterium* strain GV3101. To establish VIGS, cotyledons of 10-days-old plants were agroinfiltrated with a mixture of TRV1 and TRV-U196237 (combined in a 1: 1 ratio). As a control, a mixture of TRV1 and empty TRV2 vector (TRV-EV) was used. Three weeks after agroinfiltration the plants were sprayed with *On* inoculum. Three independent experiments were performed. For two experiments disease symptoms were visually scored 21 dpi by counting the number of fungal colonies on the leaves. For one experiment silencing level and fungal growth were quantified by qRT-PCR, using RNA from five plants for TRV-EV and 10 plants for TRV-U196237.

### Generation of stable silenced lines

To suppress both *ALS1* and *ALS2* by RNAi, the same fragment as used in VIGS construct TRV-U196237 was introduced into the pHellsgate8 vector [44]. For the purpose of targeting *ALS1* and *ALS2* separately, primers were designed based on the 3’ UTR sequences. For *ALS1*: Fw-*ALS1*-caccGCCAAAAGTGTTCGATTTGT and Rv-*ALS1*-AGTGAACATAAATACCAAGTAGAAGAT. For ALS2: Fw-*ALS2*-caccTGTTTACTTAAAAGTTTTTC ATTGTG and Rv-*ALS2*-TTAGTCATACTAAATAGAGCTCCAAA. To suppress *ALS3*, primers were designed based on the sequences in coding region: Fw-*ALS3*-caccTTATCTTGGAAATCCTTCTAACAA and Rv-*ALS3*-TTCTTATGAATCACTTGAGCA. Fragments amplified with abovementioned primers were introduced into pHellsgate8 vector [40] and finally transformed into *Agrobacterium* strain AGL1 + virG. For generation of silenced lines the protocol described by Huibers et al. [10] was used. Primary transformants (T1) were selfed to produce T2 families. For each segregating T2 family, CaMV 35S promoter primers Xho-Fw-TGCTGACCCACAGATGGTTA and 35S2-GATAGTGGGATTGTGCGTCA [45] amplifying a 756-bp fragment, or NPTII primers Fw-NPTII-TTCCCCTCGGTATCCAATTA and Rv-NPTII-GATTGTCTGTTGTGCCCAGT amplifying a 170-bp fragment from the pHellsgate8 T-DNA, were used to select transgenic T2 plants.

### Herbicide application

Chlorsulfuron was purchased from Aldrich-Sigma (PS-1065), and the powder was dissolved in acetone (0.2 mg/ml). The herbicide solution was applied to 30-day-old plants of MM and NIL-*Ol-1* growing in ø14 cm pots in normal potting soil. Before application, watering was suspended for two days to ensure that the solution could be absorbed completely. As controls, water and acetone were applied. The chlorsulfuron solution, acetone and water were added to the soil with a pipette (8 ml per pot). After this, the plants were challenged with powdery mildew *On* at the same day of herbicide application. Per genotype 15 plants were treated with chlorsulfuron, and five plants with water or acetone.

### Quantitative reverse transcription PCR (qRT-PCR) and data analysis

For quantification of fungal biomass, DNA or RNA extracted from tomato leaves was used. For quantification of transcript levels, RNA was used. DNA was isolated with DNeasy plant mini kit (Qiagen). Total RNA was extracted from leaflets using the RNeasy kit (Qiagen). After removal of DNA with DNase I (Invitrogen), 1 μg total RNA was used for cDNA synthesis using SuperScript II Reverse Transcriptase kit (Invitrogen). Quantitative real-time PCR was conducted using the iQ SYBR Green supermix (Bio-Rad) and the CFX96 Real-Time system (Bio-Rad). The PCR amplification consisted of an initial denaturation step of 3 min at 95°C, followed by denaturation for 15 sec at 95°C, annealing and extension for 1 min at 60°C for 39 cycles, then a final melt step from 65°C to 95°C ramp with 0.5°C increments per cycle to monitor specificity. Primers used for fungal quantification were Fw-*On*-CGCCAAAGACCTAACCAAAA and Rv-*On*-AGCCAAGAGATCCGTTGTTG. Primers for tomato *elongation factor 1α* (*EF*) were Fw-*EF*-GGAACTTGAGAAGGAGCCTAAG and Rv-*EF*-CAACACCAACAGCAACAGTCT [46]. For detection of relative transcript levels of the *ALS* genes primers were Fw-*ALS1*-CGCTCAACATAATCGTCGTG and Rv-*ALS1*-ACGGGAAACGAATGTTTCAG for *ALS1*; Fw-*ALS2*-CCCTTCTTCCCAAATCTACCT and Rv-*ALS2*-TTGAAACAGTGAAACGGCTATG for *ALS2*; Fw-*ALS3*-TTTGCTGCTAGCATTTGGAG and Rv-*ALS3*- GGAGTCGATATCAATGTGAACAA for *ALS3*. For the time-course experiment in which the expression of three *ALS* genes was monitored after inoculation with *On*, the same set of primers was used as for detection of relative transcript levels of each *ALS* gene after silencing. For analysis of the relative expression level and fungal biomass the 2^-ΔΔCt^ method as described by Livak and Schmittgen [47] was used. Data were statistically examined using independent-samples t-test, one-way analysis of variance or two-way between groups ANOVA based on Post-hoc comparisons using Tukey’s HSD test (*P* <0.05). All analyses were performed using SPSS Statistics 20 following the instructions of SPSS Survival Manual 4^th^ edition [48].

## Competing interests

The authors declare that they have no competing interests.

## Authors’ contributions

DG carried out the experiments. AEHML participated in the herbicide experiment. DG, RPH, and YB designed the experiments. YB drafted the outline of the manuscript, and DG and AMAW wrote the paper. YB and RGFV commented on the manuscript. All authors read and approved the final manuscript.

## Supplementary Material

Additional file 1 **Sequence alignments. (A)**, Sequence alignment of TDF M11E69-195 and *ALS* PCR fragment used in VIGS and RNAi constructs ALS1 + 2 (both from NIL-*Ol-1*) with the corresponding part of unigene SGN-U196237 from *Capsicum annuum*, and of *ALS1*, *ALS2* and *ALS3* transcripts from tomato cultivar Heinz. Nucleotides identical with the *ALS3* sequence are highlighted. **(B)**, Sequence alignment of *ALS1*, *ALS2* and *ALS3* coding sequences (CDS) of tomato cultivar Heinz, and the *ALS3* CDS of NIL-*Ol-1*. Nucleotides identical with the Heinz *ALS3* sequence are highlighted. **(C)**, Sequence alignment of protein sequences from tomato cultivar Heinz ALS1, ALS2 and ALS3 with ALS3 from NIL-*Ol-1*. Amino acids identical with the ALS3 sequence are highlighted. Click here for file

Additional file 2 **Absence of cross-silencing by RNAi constructs targeting individual *ALS *** **genes.** Cross-silencing was not detected in three representative silenced lines in each of which a specific *ALS* gene was targeted by RNAi (RNAi-ALS1, 2 and 3). Values were normalized relative to *EF*, and calibrated to the levels in untransformed NIL-Ol-1 plants. Error bars represent standard deviation of three biological replicates. Click here for file

Additional file 3 **RNA-seq data of *ALS1, ALS2 *** **and *ALS3 *** **genes.** Expression level of *ALS1*, *ALS2* and *ALS3* genes from tomato, *S. pimpinellifolium* and potato in leaves and root (for tomato) or tuber (for potato) derived from RNA-seq data, and indicated as Fragments per Kilobase of exon per Million fragments mapped (FPKM) or Reads per Kilobase of transcripts per Million mapped (RPKM) values. Click here for file

Additional file 4 **Effect of exogenous application of amino acids on *Oidium neolycopersici *** **(*On*) fungal growth.** Quantification of *On* fungal biomass, 8 days post inoculation of NIL-*Ol-1* plants sprayed with different amino acids solutions. Amino acids homoserine (HS), threonine (Thr), or branched-chain amino acids valine (Val), isoleucine (Ile) or leucine (Leu) were applied as described in Huibers et al. [10]. Data indicate the mean of three biological replicates with error bars representing the standard deviation. The asterisk indicates significant difference from the H_2_O control according to one way analysis of variance (*P* <0.05). Click here for file
