# Supplementary figures and images for: Down-regulation of acetolactate synthase compromises Ol-1- mediated resistance to powdery mildew in tomato
Source: BMC Plant Biol. 2014 Jan 17;14:32. doi: 10.1186/1471-2229-14-32 (PMC3898995; doi:10.1186/1471-2229-14-32)

**Additional file 1A**


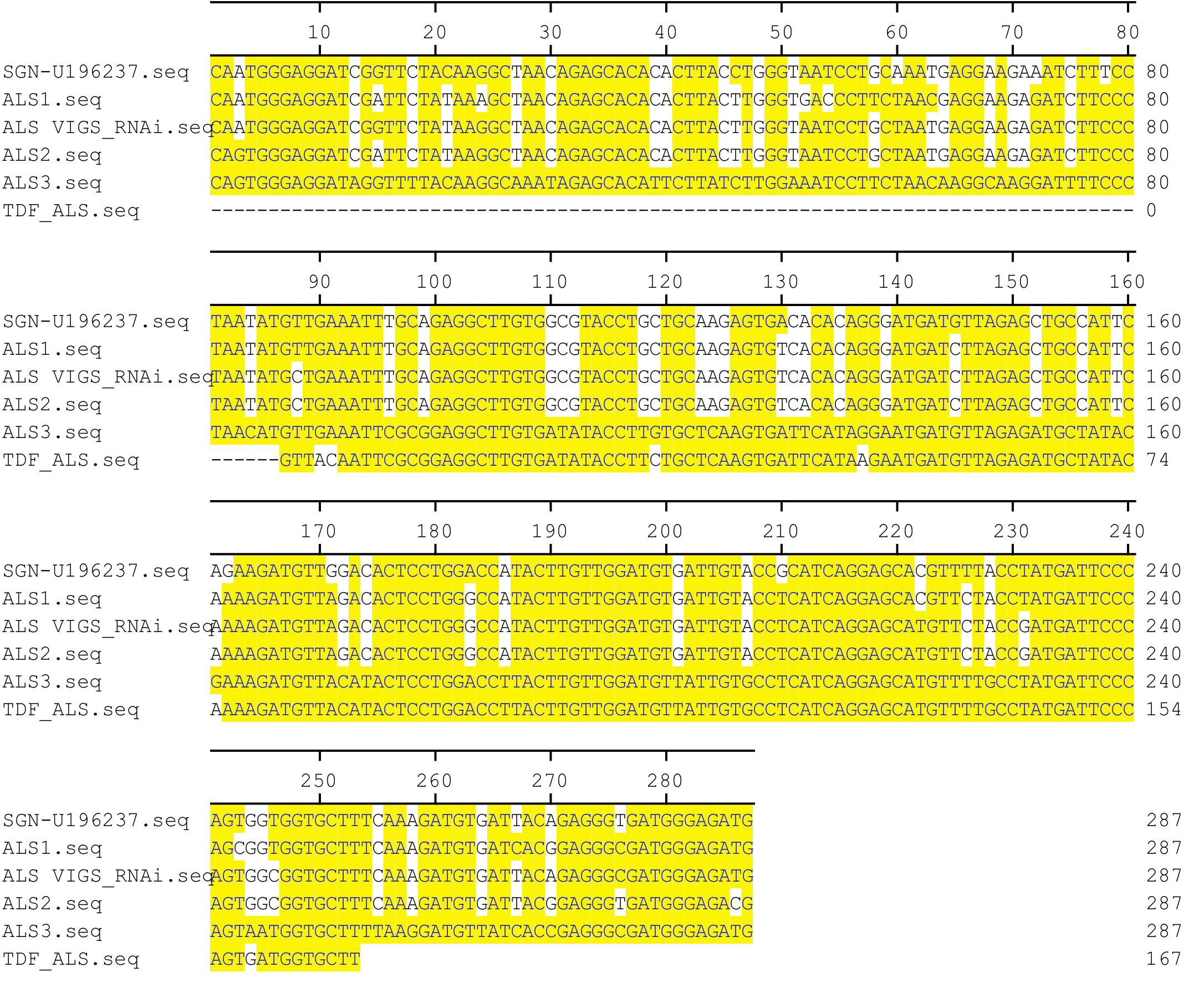


**Additional file 1B**
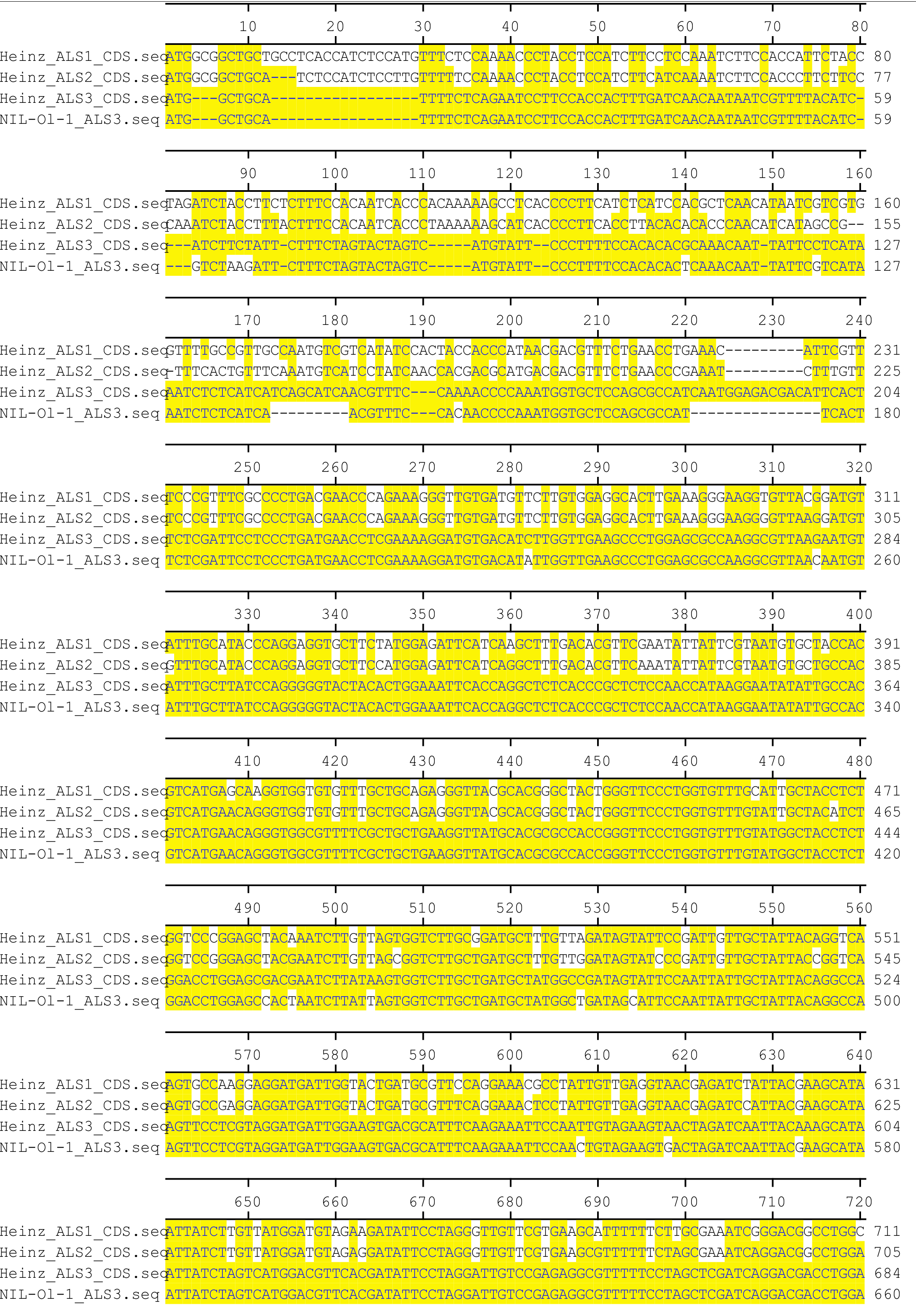

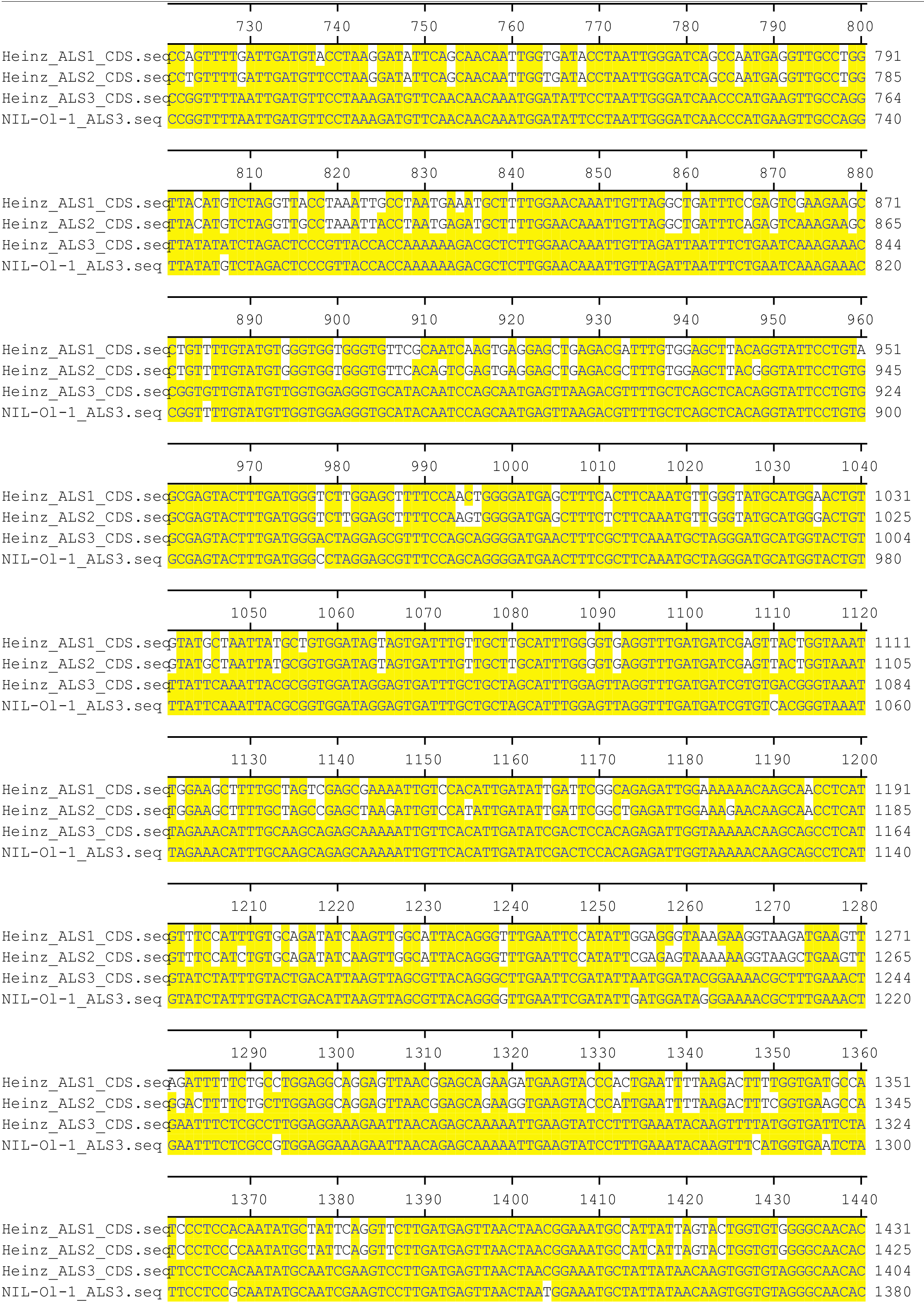

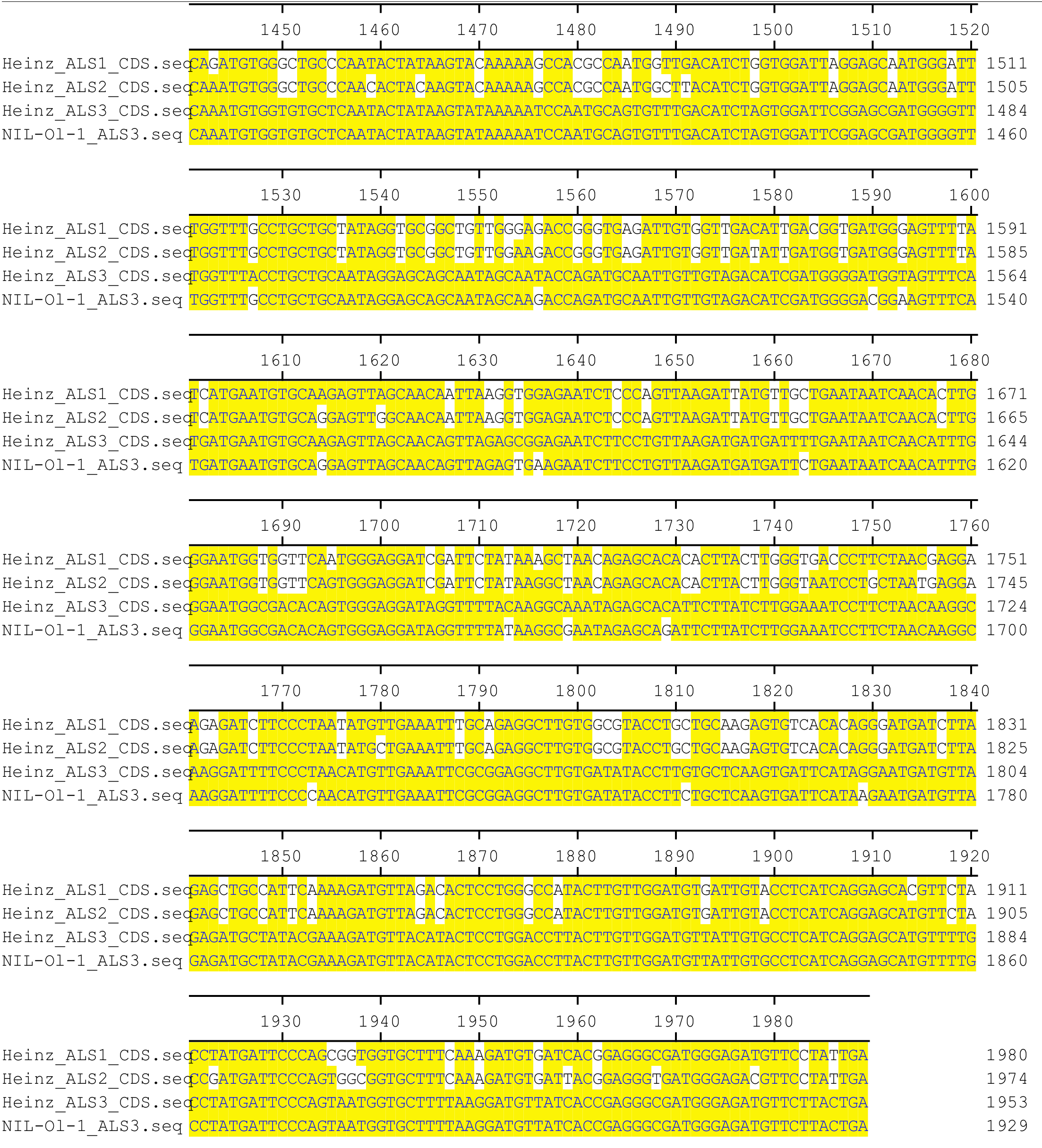


**Additional file 1C**


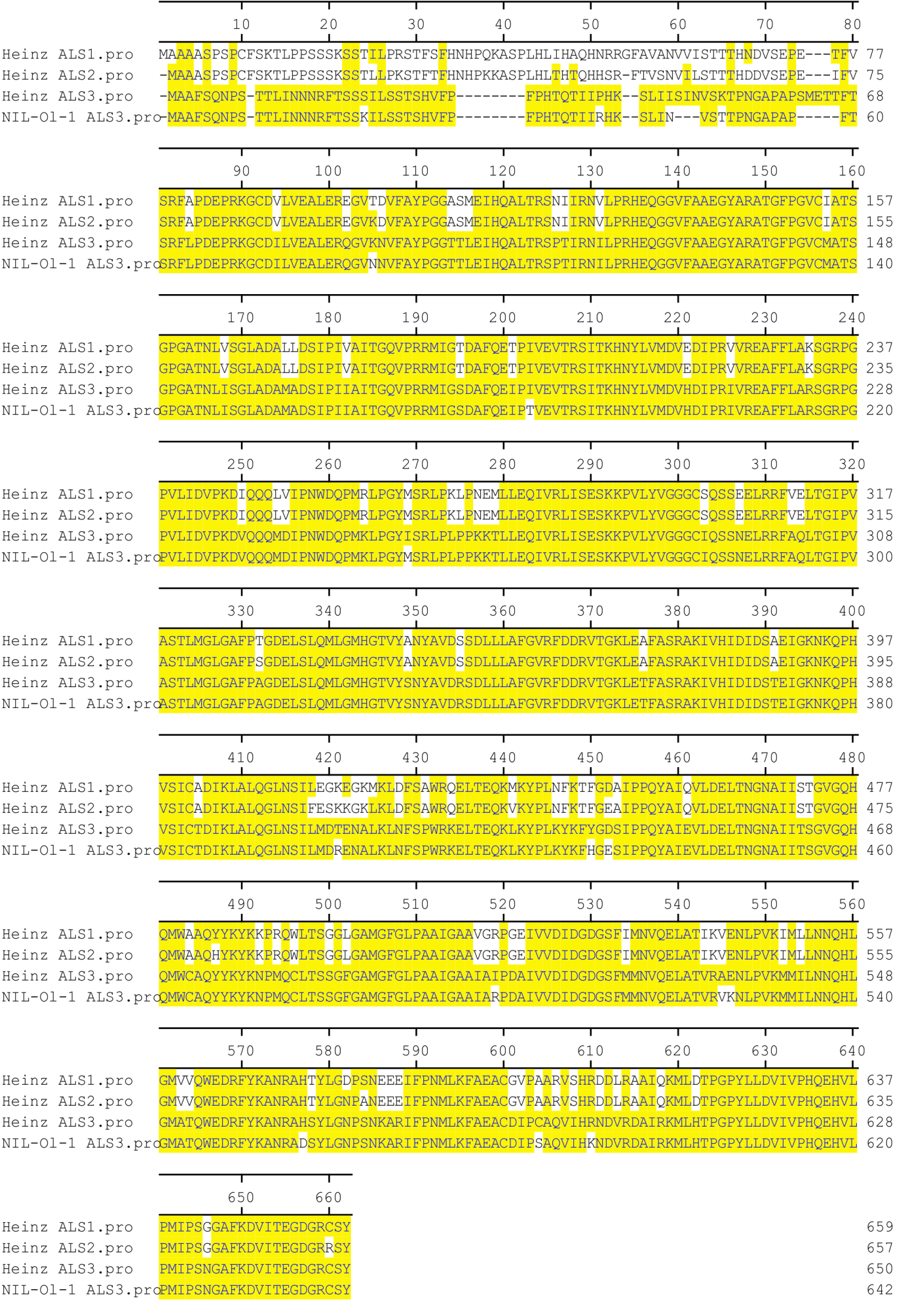

Supplement: Additional file 1 — Sequence alignments. (A), Sequence alignment of TDF M11E69-195 and ALS PCR fragment used in VIGS and RNAi constructs ALS1 + 2 (both from NIL-Ol-1) with the corresponding part of unigene SGN-U196237 from Capsicum annuum, and of ALS1, ALS2 and ALS3 transcripts from tomato cultivar Heinz. Nucleotides identical with the ALS3 sequence are highlighted. (B), Sequence alignment of ALS1, ALS2 and ALS3 coding sequences (CDS) of tomato cultivar Heinz, and the ALS3 CDS of NIL-Ol-1. Nucleotides identical with the Heinz ALS3 sequence are highlighted. (C), Sequence alignment of protein sequences from tomato cultivar Heinz ALS1, ALS2 and ALS3 with ALS3 from NIL-Ol-1. Amino acids identical with the ALS3 sequence are highlighted. [file 1471-2229-14-32-S1.doc]

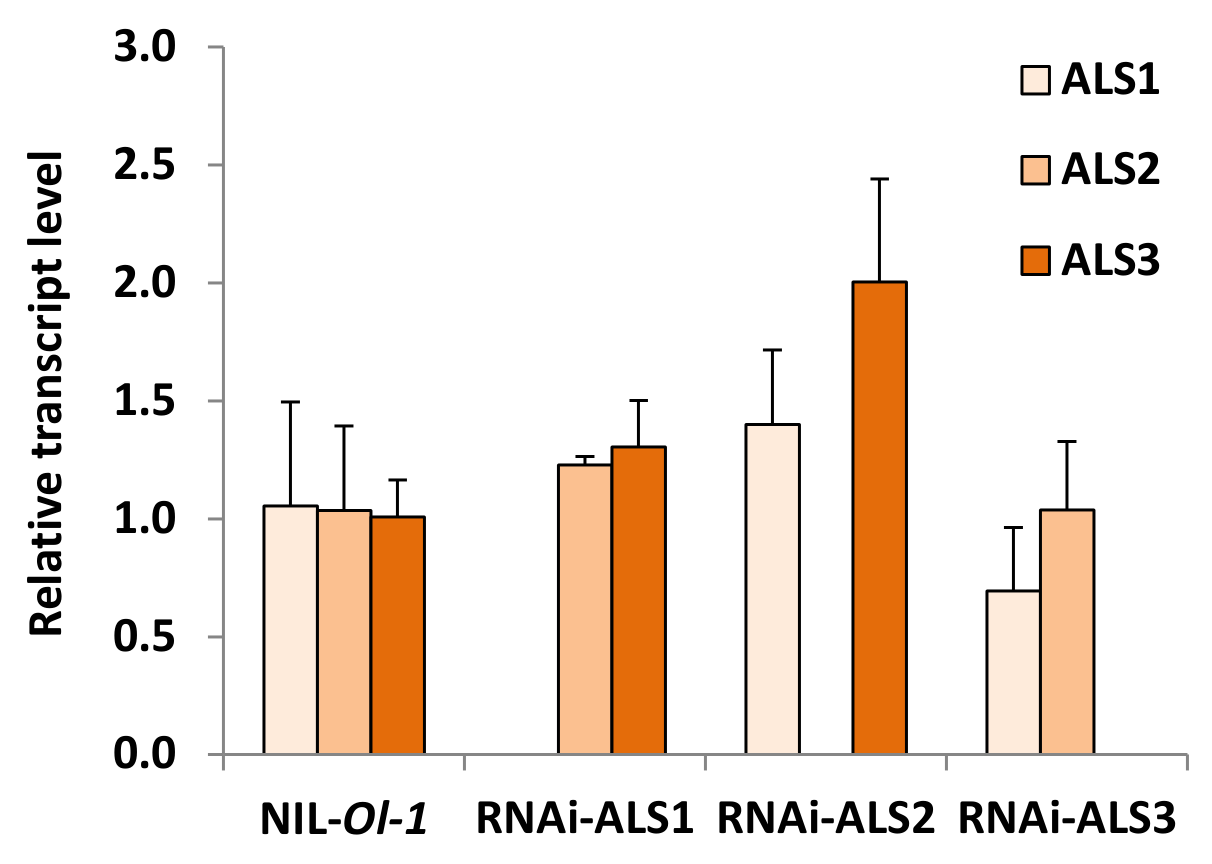

Supplement: Additional file 2 — Absence of cross-silencing by RNAi constructs targeting individual ALS genes. Cross-silencing was not detected in three representative silenced lines in each of which a specific ALS gene was targeted by RNAi (RNAi-ALS1, 2 and 3). Values were normalized relative to EF, and calibrated to the levels in untransformed NIL-Ol-1 plants. Error bars represent standard deviation of three biological replicates. [file 1471-2229-14-32-S2.png]

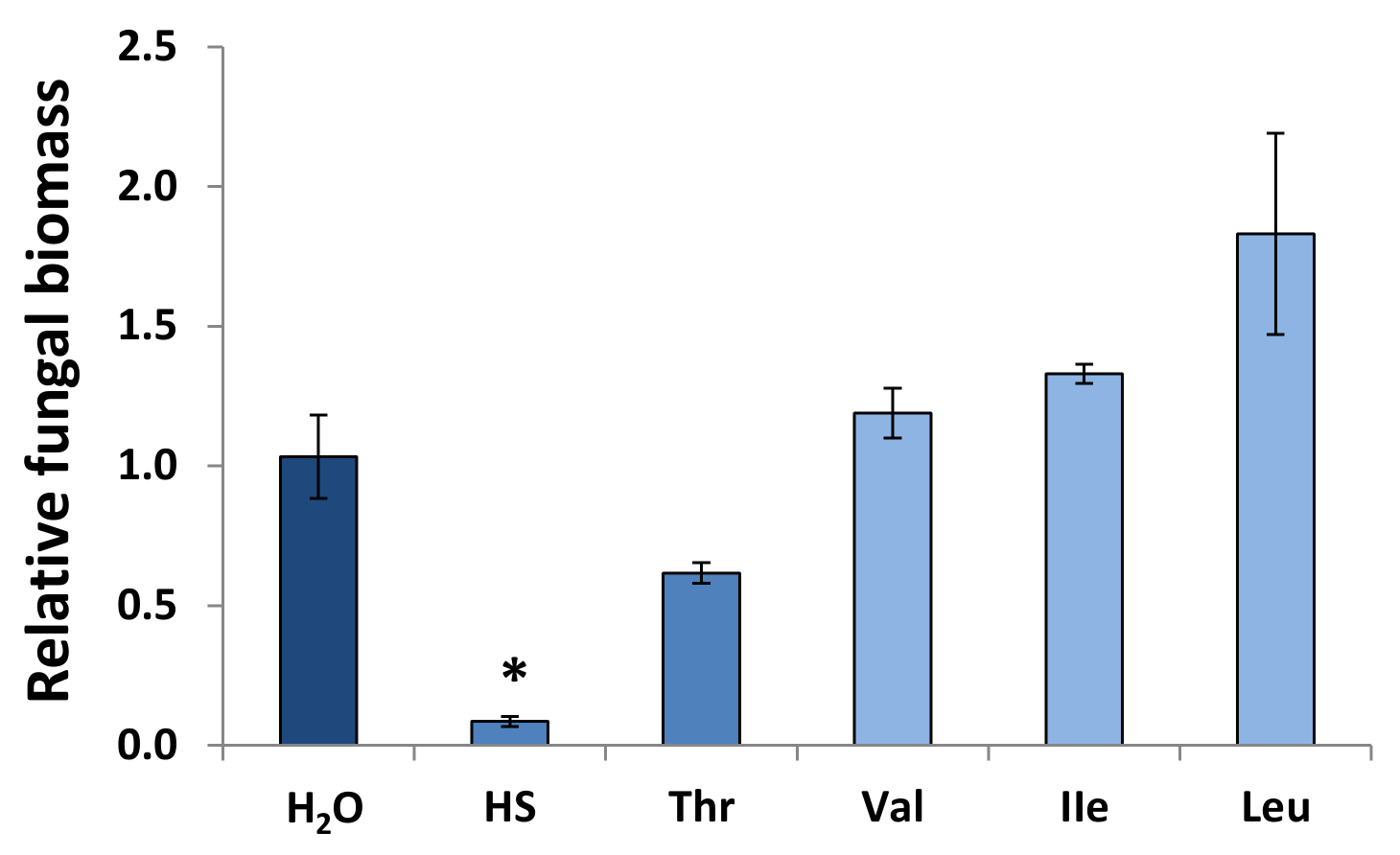

Supplement: Additional file 4 — Effect of exogenous application of amino acids on Oidium neolycopersici ( On ) fungal growth. Quantification of On fungal biomass, 8 days post inoculation of NIL-Ol-1 plants sprayed with different amino acids solutions. Amino acids homoserine (HS), threonine (Thr), or branched-chain amino acids valine (Val), isoleucine (Ile) or leucine (Leu) were applied as described in Huibers et al. [10]. Data indicate the mean of three biological replicates with error bars representing the standard deviation. The asterisk indicates significant difference from the H2O control according to one way analysis of variance (P <0.05). [file 1471-2229-14-32-S4.png]
